# The Diverse Consequences of *FOXC1* Deregulation in Cancer

**DOI:** 10.3390/cancers11020184

**Published:** 2019-02-05

**Authors:** L. Niall Gilding, Tim C. P. Somervaille

**Affiliations:** Leukaemia Biology Laboratory, Cancer Research UK Manchester Institute, The University of Manchester, Manchester M20 4JG, UK; niall.gilding@cruk.manchester.ac.uk

**Keywords:** epigenetics, transcription factor, epithelial-mesenchymal transition, metastasis, cellular reprogramming, pioneer factor

## Abstract

Forkhead box C1 (FOXC1) is a transcription factor with essential roles in mesenchymal lineage specification and organ development during normal embryogenesis. In keeping with these developmental properties, mutations that impair the activity of FOXC1 result in the heritable Axenfeld-Rieger Syndrome and other congenital disorders. Crucially, gain of FOXC1 function is emerging as a recurrent feature of malignancy; FOXC1 overexpression is now documented in more than 16 cancer types, often in association with an unfavorable prognosis. This review explores current evidence for FOXC1 deregulation in cancer and the putative mechanisms by which FOXC1 confers its oncogenic effects.

## 1. FOXC1 Structure and Function

**Figure 1:**
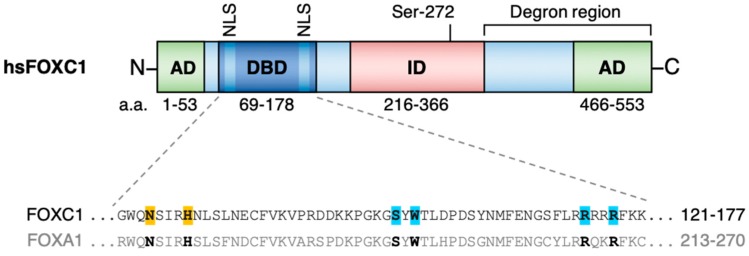
(**Above**) Overview of FOXC1 protein structure and functional protein domains identified by in vitro analyses. (**Below**) Amino acid sequence alignment of critical residues in the Forkhead domain of FOXC1 which are essential for the DNA-binding properties of FOX proteins. Residues highlighted in orange are indispensable for sequence-specific recognition of the FOX DNA motif, while those highlighted in blue promote non-specific engagement of nucleosomal DNA by FOXA proteins, consistent with pioneer activity [10,11]. AD, activating domain; DBD, Forkhead DNA-binding domain; ID, inhibitory domain; NLS, nuclear localization signal; *hs, Homo sapiens*.

*FOXC1* (previously referred to as FREAC3 or FKHL7) is a single exon gene mapping to chromosome 6p25.3 in humans which encodes a single isoform 553 amino acid protein [1,2] (Figure 1). Like the other ~50 members of the human Forkhead family, FOXC1 contains a highly conserved ~110 amino acid Forkhead DNA binding domain. The Forkhead domain comprises 4 N-terminal α-helices, 3 β-strands and 2 C-terminal wings which collectively form a ‘winged helix’ structure for interaction with DNA via the consensus DNA motif 5′-TGTTTAC-3′ [3,4]. In the case of FOXC1 specifically, the Forkhead domain is demarcated by two nuclear localization signals for retention of FOXC1 protein in the nucleus [5]. The Forkhead domain is unique among DNA-binding domains in that its structure is similar to that of the linker histones H1 and H5 (which are involved in generation of a compacted higher order chromatin structure). This endows Forkhead family members (e.g., FOXA, FOXO and FOXE) with a specialized ability to directly engage DNA in compacted chromatin [6,7,8,9] allowing so-called pioneer activity: the ‘scanning’ of compacted chromatin, identification of target binding sites and direct promotion of nucleosome destabilization to allow other non-pioneer transcription factors to bind to consensus sites nearby. Indeed, FOXC1 exhibits conservation of the critical amino acids which confer pioneer activity in the Forkhead domain of FOXA1 [10,11,12]. Thus, although FOXC1 has not itself been formally confirmed as a pioneer factor, it seems probable it is one. Pioneer transcription factors prime the transition of chromatin from a condensed, inactive state to an accessible, transcriptionally competent one and are essential for the correct spatiotemporal regulation of genes in development, mitosis and adult cell-state transitions [13]. Pioneer transcription factor activity is exploited experimentally during in vitro cell reprogramming and can become hijacked during malignant transformation in concert with other transcription factors [14,15,16].

Beyond the Forkhead domain, FOXC1 shares little sequence conservation with family members outside the FOXC clade [3]. In vitro studies have found that FOXC1 has two activation domains (ADs) at the N- and C-termini which both enhance FOXC1-dependent transcription [14]. Indeed, in HeLa cells FOXC1 peptides lacking the N-terminal AD (a.a. 1–51) or C-terminal AD (a.a. 435–553) exhibited a 45% or 60% respective reduction in activity of a luciferase reporter relative to intact FOXC1 [17], and combined deletion of both ADs resulted in an 80% reduction. Consistent with these findings, fusion of either AD to a GAL4 DNA-binding domain greatly increased the activity of a GAL4-responsive luciferase reporter gene in COS-7 cells, underlining the *trans*-activating potential of these two domains.

The same studies also identified an inhibitory domain (ID) (a.a. 215–366). In contrast to the results observed following AD removal, expression of a truncated version of FOXC1 lacking the ID increased luciferase reporter activity in HeLa cells by 40% compared to full-length FOXC1 [17]. The ID contains a phosphorylation site at S272 which directly influences FOXC1 protein stability in vitro [18]. Phosphorylation of S272 by ERK1/2 prolongs the half-life and transcriptional output of FOXC1 protein in HeLa cells, whereas a non-phosphorylatable S272A mutant FOXC1 protein is rapidly targeted for degradation by the 26S proteasome [18]. Based on distinct trypsin digestion patterns of recombinant FOXC1 following treatment with phosphatases, it is thought that phosphorylation of sites, including S272, impair the ability of proteases to recognize cleavage sites within the FOXC1 degron region. Finally, FOXC1 also contains two motifs which can be covalently linked to small ubiquitin-like modifier (SUMO) proteins. In one study, SUMOylation of FOXC1 was found to inhibit its transcriptional activity without affecting subcellular localization or protein stability [19]. The authors of this study also documented additional putative phosphorylation sites within the murine FOXC1 ID, although the functional significance of these sites is less well understood.

## 2. FOXC1 in Tissue Development, Homeostasis and Disease

### 2.1. FOXC1 and Development

As for other Forkhead proteins, FOXC1 is a critical regulator of lineage specification and its expression is highly regulated throughout development. *Foxc1* is initially upregulated in neural crest cells, promoting an epithelial-mesenchymal transition (EMT) required for correct neural tube formation [20]. As development proceeds its expression is associated with somite formation and the emergence of bone and cartilage from osteogenic and chondrogenic mesenchyme, respectively [21,22]. Later on, expression in distinct mesenchymal settings promotes the development of other tissues and organs, including the anterior eye segments, hindbrain, cardiovascular and urinary systems [2,23,24,25]. The essential roles of *Foxc1* expression throughout development are highlighted by knockout studies; *Foxc1*-null mice typically die in utero or neonatally with hydrocephaly and skeletal, cardiac, renal and ocular defects [21,23,24,25]. Consistent with murine knockout phenotypes, impaired FOXC1 function is associated with developmental malformations in humans. These include Axenfeld-Rieger syndrome, a group of autosomal dominant conditions characterized by defects in the anterior chamber of the eye together with variable skeletal, cardiac and craniofacial abnormalities. Axenfeld-Rieger syndrome (ARS) patients commonly feature deletion or mutation of one *FOXC1* allele, and 31 distinct point mutations have been identified in *FOXC1* in association with ARS to date. Twenty-nine of these occur within the Forkhead domain of FOXC1 and typically impair DNA binding, nuclear localization or protein stability [5]. The diversity of *FOXC1* mutations is thought to account for the range of clinical manifestations of ARS [26,27,28]. More recently, *FOXC1* mutations were linked to Dandy-Walker syndrome, a group of disorders characterized by cerebellar defects and a variable set of craniofacial, cardiac and limb abnormalities [29,30]. Another report found that *FOXC1* mutations were also associated with diverse microvascular abnormalities in the brain consistent with cerebral small vessel disease [31]. Collectively, these observations highlight a range of critical developmental processes supported by correct expression of *FOXC1*.

### 2.2. FOXC1 Contributes to the Maintenance of Adult Stem and Progenitor Cell Compartments

Beyond the essential contributions made by FOXC1 during prenatal development, more recent studies have identified additional roles for FOXC1 in the regulation of adult stem cells. One of the most well-characterized examples is that of hair follicle stem cells where *Foxc1* expression is enriched by comparison with downstream more differentiated hair follicle cells [32]. In collaboration with NFATC1, FOXC1 upregulates genes promoting quiescence, restraining the rate of stem cell activation to ensure sustained hair growth throughout life. Conditional ablation of FOXC1 in stem cells led to increased cycling, premature exhaustion and progressive hair loss [33]. Elsewhere, FOXC1 was found to restrict the differentiation of dental pulp stem cells into the cells responsible for tooth mineralization [34] possibly through suppression of pro-odontogenic signaling pathways. In the mammary gland, FOXC1 is highly expressed in the clonogenic CD44^+^ compartment enriched for luminal progenitor cells [35,36,37] and its forced expression in luminal epithelium-like MCF12A cells induced a less-differentiated CD44^+^ mesenchymal-like phenotype [35]. Consistent with these findings, forced expression of *FOXC1* in the murine mammary gland was associated with expansion of the luminal progenitor compartment, impaired lobuloalveolar development and an ultimate failure of lactation [38]. Thus, appropriate levels of *FOXC1* expression appear to be crucial for correct development and function of the mammary gland. In summary, despite limited insights into the specific mechanisms and pathways involved, FOXC1 appears to be an important cell-intrinsic regulator of adult stem and progenitor cell function in certain tissues.

Interestingly, an indirect role for FOXC1 in the maintenance of adult hematopoietic stem cells was recently reported. Nagasawa and colleagues found that *Foxc1* expression in CXCL12-abundant reticular cells in murine bone marrow is required to maintain the niche where adult hematopoietic stem cells reside [39]. Indeed, selective ablation of *Foxc1* in niche cells in utero led to a depletion of both niche cells and hematopoietic stem cells inducing a collapse of mature blood cell counts. As FOXC1 is not expressed in normal hematopoietic cells [40], these observations highlight an essential, cell-extrinsic mechanism through which FOXC1 supports normal hematopoiesis.

### 2.3. FOXC1 Overexpression is a Frequent and Functional Event in Cancer

In contrast to non-malignant disorders such as Axenfeld-Rieger syndrome and Dandy-Walker syndrome—which are characterized by reduced normal FOXC1 function—the opposite appears generally true in cancer. Overexpression of *FOXC1* has been reported in at least 16 types of cancer, often in association with a poor prognosis (Table 1). This point is further emphasized by the lack of hypomorphic *FOXC1* mutations observed in human malignancy. However, although overexpression of *FOXC1* is frequent, the mechanisms of *FOXC1* deregulation and how these influence oncogenic processes seem specific to each tumor setting.

## 3. FOXC1 Action Across the Hallmarks of Cancer

It is now appreciated that cancer cells typically acquire several biological properties that facilitate tumor progression. Although the specific molecular processes vary greatly from one cancer type to another, the unifying consequences can be organized into discrete ‘hallmarks’ of cancer initiation and progression [66]. Crucially, deregulation of FOXC1 contributes to several of these hallmarks across various tumor settings. These include an increased capacity for proliferation, resistance to apoptosis and/or drug therapy, adaptation to hypoxia and enhanced cellular invasion/migration. In this section, the current evidence relating FOXC1 to the hallmarks of cancer will be explored.

### 3.1. FOXC1 Enhances Cancer Cell Proliferation and Survival in Diverse Cancer Types

Cancer cells are typically endowed with an increased capacity for survival and proliferation [66]. Several lines of evidence indicate that overexpression of FOXC1 contributes to these processes, perhaps most notably in studies of acute myeloid leukemia (AML) and basal-like breast cancer (BLBC).

In AML, there are several leukemogenic consequences of aberrant de-repression of *FOXC1*, which occurs in fully ~20% of human AML cases, typically in association with concomitant high-level *HOXA/B* expression [40]. Knockdown of *FOXC1* in AML cell lines diminished clonogenic potential by inducing a G1 cell-cycle arrest, morphologic differentiation and apoptosis. By contrast, forced expression of FOXC1 in normal murine CD117^+^ bone marrow stem and progenitor cells promoted a transient block to the normal differentiation of the myeloid progenitor compartment and enhanced clonogenic activity. Critically, forced expression of *FOXC1* and *Hoxa9* in stem and progenitor cells generated an aggressive, serially-transplantable AML. Compared to AMLs arising from expression of *Hoxa9* alone, murine *FOXC1*^hi^*Hoxa9*^hi^ AMLs had shortened latencies, a higher proportion of actively dividing leukemia cells and a block to monocyte/macrophage lineage differentiation. Analysis of transcriptome data from a large cohort of AML patients revealed that blocked monocytic lineage differentiation is also a feature of human FOXC1^hi^ AML providing further evidence that high level FOXC1 expression is functionally important. The monocyte lineage transcription factor *KLF4* emerged as a critical FOXC1-repressed target, since restoration of *KLF4* expression was sufficient to release the differentiation block in AML cell lines with high *FOXC1* expression. Consistent with these findings, *KLF4* is upregulated in *FOXC1*-expressing AML cell lines induced to differentiate pharmacologically, as *FOXC1* expression is repressed in parallel. Thus, in human AML, FOXC1 collaborates with high HOXA/B expression to promote disease. Indeed, the proliferation-enhancing properties of FOXC1 in AML were recently corroborated in primary human AML samples; as in earlier studies of AML cell lines, there was a substantial loss of clonogenic potential following *FOXC1* knockdown [67]. Interestingly, another study of primary AMLs with internal tandem duplication of *FLT3* (*FLT3*-ITD) found that *FOXC1* upregulation was associated with a global redistribution of genome occupancy by normal myeloid TFs including C/EBP, PU.1 and RUNX proteins [68]. Taken together, these studies suggest that FOXC1 blocks myeloid differentiation by reprogramming the epigenetic landscape of AML cells. However, it is not yet clear whether FOXC1 achieves this by co-opting normal myeloid transcription factor regulatory networks or instead via direct repression of monocyte transcriptional regulators such as *KLF4*. Genome-wide FOXC1 occupancy data are needed to identify direct target genes of FOXC1 and disease-specific mechanisms that may be targeted for therapeutic gain.

In the context of BLBC, *FOXC1* knockdown in various breast cancer cell lines suppressed proliferation and induced more differentiated cellular morphology [42,69]. Conversely, forced expression of *FOXC1* in MCF-7 breast cancer cells significantly enhanced their proliferation and anchorage-independent growth in vitro [42]. In another study, forced expression of FOXC1 in a panel of breast cancer cell lines was accompanied by an increase in levels of cyclin D1 and c-Myc [69]. Indeed, both of these putative FOXC1 targets are proto-oncogenes which contribute to cell cycle progression and are commonly deregulated in cancer [70,71]. Interestingly, cyclin D1 expression is also induced in a FOXC1-dependent manner in models of pancreatic and non-small cell lung, promoting cell survival and proliferation [61,72].

Collectively, these data strongly indicate that FOXC1 enhances cell proliferation and alters cell fate across diverse cancer types. However, with the notable exception of cyclin D1, the specific downstream targets of FOXC1 which sustain aberrant proliferation in cancer remain largely unidentified.

### 3.2. FOXC1 As a Putative Regulator of Cancer Stem Cell Function

Cancer stem cells exist at the apex of the tumor clonal hierarchy, sustain tumor growth and provide a reservoir for relapse following treatment [73]. Studies suggest that FOXC1 may modulate the activity of these cells. For instance, overexpression of *FOXC1* in breast cancer cells increased the proportion of cells expressing the putative stem cell markers ALDH and CD133, and enhanced mammosphere formation in vitro [44]. In the same study, FOXC1 enhanced the DNA-binding activity of GLI2, a member of the Hedgehog signaling pathway reported elsewhere as preferentially activated in cancer stem cells [74]. In analyses of large datasets of breast cancer patient gene expression, *FOXC1* expression correlated positively with increasing activation of an Hh gene signature and lower disease-free survival [44]. Crucially, forced expression of *FOXC1* rendered breast cancer cells resistant to pharmacologic inhibition of Smoothened, a membrane-bound receptor involved in canonical Hedgehog signaling. Consequently, the authors proposed that FOXC1-dependent activation of non-canonical Hedgehog signaling is a potentially significant contributor to CSC-like properties in breast cancers with high FOXC1 expression. Interestingly, the same laboratory recently reported in unrelated studies of triple negative breast cancer FOXC1-dependent activation of non-canonical WNT signaling, a pathway also thought to contribute to CSC phenotypes [74,75].

In non-small cell lung cancer (NSCLC) cells, knockdown of *FOXC1* impaired self-renewal, downregulated CD133 and other ‘stemness’ markers, dampened in vivo tumorigenicity, and increased sensitization to chemotherapy [56]. Using DNA-binding assays, the authors posited that FOXC1 enhances cancer stem cell activity by upregulating β-catenin, the transcriptional mediator of WNT signaling. Indeed, the stem-like characteristics of NSCLC cells with high levels of FOXC1 were lost following knockdown of β-catenin. Interestingly, the emerging evidence of a mechanistic interplay between CSC signaling pathways such as Hedgehog and WNT is consistent with studies of the normal developmental regulation of Forkhead family transcription factors. In these contexts, the expression of individual FOX genes is generally thought to be controlled by the integration of several developmental pathways, including Hedgehog and others [76].

### 3.3. FOXC1 Expression Enhances the Adaptation to Tumor Hypoxia

Hypoxia is a common feature of rapidly-growing tumors lacking a sufficient blood supply, and stimulates adaptations to promote cell survival including angiogenesis, metabolic changes and enhanced stem-like properties [77]. Central to the hypoxic response are the Hypoxia Inducible Factor (HIF) family of transcription factors which regulate the transcription of stress-response genes. Critically, *FOXC1* appears to be a downstream target of HIF signaling. Studies in lung cancer cell lines have demonstrated that *FOXC1* transcription is directly upregulated by HIF-1α, and its expression is enriched in hypoxic regions of primary lung tumors [77]. Consistent with an important role in the hypoxic adaptation of lung tumors, hypoxia-inducible knockdown of *FOXC1* blunted tumor growth, angiogenesis and metastasis in vivo. Similarly, *FOXC1* was found to be upregulated by HIF-1α in studies of hepatocellular carcinoma cells; FOXC1 consequently upregulates IL-8 and other inflammatory mediators [78]. Interestingly, another study reported that FOXC1 increases the rate of anerobic glycolysis in colorectal cancer cells, perhaps due to lower oxygen availability [45]. Indeed, *FOXC1* knockdown in this setting decreased glycolysis pathway activity, proliferation and colony formation, although the specific contribution of hypoxia to these processes was not investigated. In another study in hepatocellular carcinoma, *FOXC1* knockdown led to a downregulation of the pro-angiogenic factor VEGFA, consistent with the developmental roles of FOXC1 as a VEGF-responsive mediator of blood vessel formation [24,79].

To summarize, a consistent set of studies suggests that FOXC1 may play significant roles in overcoming the deleterious effects of hypoxia to sustain tumor progression. However, the currently limited evidence base mandates further, more directed investigation to more fully understand the precise mechanisms involved.

### 3.4. FOXC1 Activates Genes Heralding the Epithelial-Mesenchymal Transition and Tumor Migration

Metastasis is a multi-step process in which cancer cells disseminate from the primary site of disease to establish tumors elsewhere in the body. The metastatic cascade is initiated when adherent tumor cells undergo an epithelial-mesenchymal transition (EMT) to allow them to detach from neighboring cells with subsequent acquisition of an invasive phenotype [80]. Following EMT, tumor cells gain characteristics which aid migration into neighboring tissues and the circulation. Crucially, it is increasingly appreciated that FOXC1 activates genes promoting both of these oncogenic processes.

Given that FOXC1 contributes to a physiologic EMT during embryonic neural tube formation, it is perhaps unsurprising that FOXC1 is also implicated with the EMT in cancer. Indeed, overexpression of *FOXC1* in low-expressing hepatocellular carcinoma cells led to downregulation of epithelial genes including E-cadherin and β-catenin in parallel with upregulation of the mesenchymal markers vimentin and fibronectin, consistent with activation of an EMT program [54]. Conversely, knockdown of *FOXC1* in high-expressing cells yielded opposite results on EMT gene expression and restored a less-invasive phenotype. Interestingly, the authors also found that FOXC1 binds the promoters of *Snail* and *Nedd9* to increase their transcription. SNAIL represses the expression of epithelial markers including E-cadherin to abrogate cell-cell adhesion, while NEDD9 enhances cellular motility in multiple cancer types [81,82]. Indeed, knockdown of *NEDD9* attenuated the invasion of *FOXC1*-overexpressing cells in vitro and in vivo, highlighting its role as a downstream mediator of FOXC1-dependent EMT in hepatocellular carcinoma. 

In studies of other cancers FOXC1 appears to promote EMT through broadly comparable mechanisms to those suggested for hepatocellular carcinoma. For instance, *FOXC1* upregulation is positively correlated with higher expression of vimentin and fibronectin, and metastasis in both nasopharyngeal carcinoma and esophageal squamous cell carcinoma [53,63]. Knockdown of *FOXC1* in either cell type led to downregulation of these mesenchymal markers, consistent with suppression of an EMT program. In a study of esophageal carcinoma cells, FOXC1 directly activated transcription of *ZEB2*, a transcriptional repressor of E-cadherin that may serve a similar role to SNAIL in hepatocellular carcinoma. Finally, FOXC1 may also underpin the EMT of breast cancer cells: *FOXC1* expression is associated with an EMT gene signature in breast circulating tumor cells, while forced expression of *FOXC1* in normal mammary epithelial cells induced a mesenchymal-like phenotype and downregulation of E-cadherin [42,83]. Interestingly, another study of luminal B breast cancer found that FOXC1 expression was associated with impaired invasion and metastasis. However, EMT marker genes were still found to be activated and the most robust FOXC1 targets identified appeared to promote a lineage-inappropriate differentiation program which the authors proposed may suppress metastasis in this specific context [43].

The process of EMT in cancer involves not just a loss of normal cell-cell adhesion but also the acquisition of invasive, motile characteristics. This was true in the aforementioned studies of hepatocellular, nasopharyngeal, esophageal and breast cancer, where *FOXC1* knockdown was accompanied by reduced invasion and migration in vitro [42,53,54,63]. However, other studies have identified additional FOXC1 targets which promote invasion in cancer, including members of the matrix metalloprotease family. These are secreted enzymes which degrade the extracellular matrix to permit cellular invasion, ordinarily during development and the wound healing response, but also in invasive cancers. Indeed, studies of breast cancer cells found that forced expression of FOXC1 induced transcription of *MMP7* [84], or *MMP2* and *MMP9* [42]. A more recent study proposed that *MMP7* upregulation occurred downstream of FOXC1-dependent activation of a WNT5A-NF-κB signaling axis in triple-negative breast cancer [75]. Overall, these findings are consistent with another study of hepatocellular carcinoma cells, where knockdown of *FOXC1* led to downregulation of *MMP1*, *2*, *7* and *9* [85]. Indeed, these observations are also in keeping with earlier reports of FOXC1-dependent induction of *MMP9* and *MMP13* during normal skeletal and microvascular development [22,79].

Interestingly, one recent study reported that alternative splicing of nuclear filamin B (FLNB)—specifically, increased generation of a short isoform lacking exon 30 (FLNB-ΔH1)—led to increased FOXC1-dependent transcription of EMT marker genes in breast epithelial-like cells [86]. Indeed, the authors demonstrated that the full-length FLNB isoform is localized to the nucleus, where it directly interacts with FOXC1 and impairs its *trans*-acting potential. FLNB-ΔH1, by contrast, is localized in the cytoplasm and is unable to exert such an effect on FOXC1, a predominantly nuclear protein. Thus, the authors proposed that increased skipping of *FLNB* exon 30 leads to reduced partitioning of FOXC1 to the less transcriptionally-active nuclear lamina, permitting induction of FOXC1 target genes promoting the EMT. More detailed investigation of this process is now needed to explore whether this is a more generalized process of FOXC1 deregulation in the EMT of other cancers.

Taken together, these studies provide compelling evidence that FOXC1 most commonly drives solid tumor progression by initiating an EMT gene signature, probably via the upregulation of mesenchymal genes driving invasion in parallel with transcriptional repressors of epithelial markers including E-cadherin. These mechanisms are entirely in keeping with the developmental roles of FOXC1 as a mesenchymal master regulator, but a lack of genome-wide FOXC1 binding data limits the identification and validation of critical target genes. Thus, it is possible that current studies have explored only a modest subset of the molecules crucial for these processes. Furthermore, the conflicting nature of some results—particularly those across different breast cancer subtypes—implies that the FOXC1-dependent transcriptional program is highly context-specific and that poorly-characterized subsets of FOXC1 target genes likely determine the differing metastatic capacities of cancer subtypes [43].

## 4. Mechanisms of FOXC1 Deregulation in Cancer

**Figure 2:**
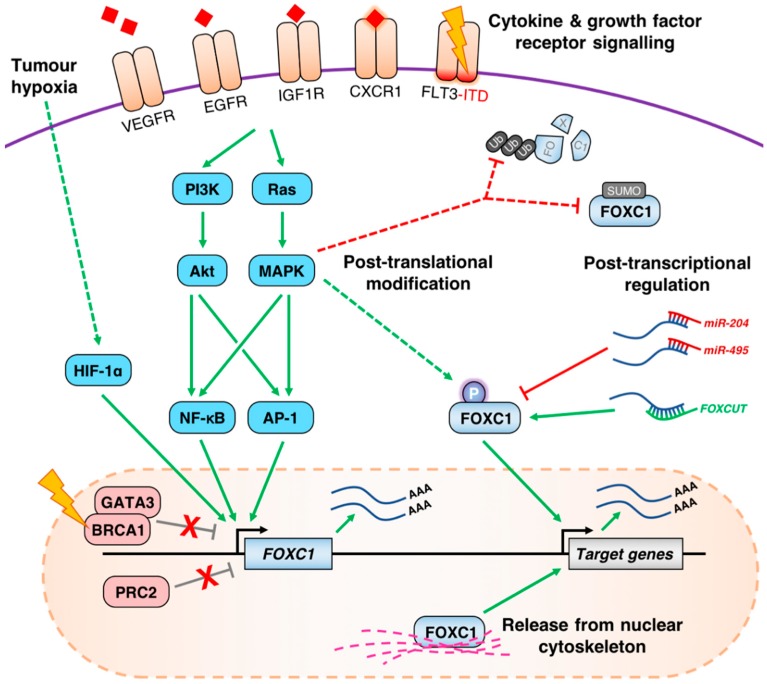
A summary of known pre-/post-transcriptional and post-translational processes regulating FOXC1 in cancer. Across several malignancies, deregulation of diverse membrane-associated receptors by mutation and/or overexpression leads to hyperactivation of the MAPK and PI3K signaling pathways and frequent induction of *FOXC1* transcription via downstream TFs including AP-1 and NF-κB. In hypoxic tumors, activation of HIF-1α may further stimulate *FOXC1* expression. These processes may occur in parallel with loss of PRC2 or BRCA1/GATA3-mediated repression of *FOXC1* through currently unclear mechanisms. *FOXC1* mRNA may be further regulated through the action of ncRNAs including *FOXCUT* and miR-204/miR-495 which enhance or impede translation into FOXC1 protein, respectively. Finally, the transcriptional activity and stability of FOXC1 protein may be modulated by partitioning in the nuclear cytoskeleton, or by post-translational modifications including phosphorylation and SUMOylation, although the contribution made by these processes to cancer-specific functionality of FOXC1 remains unclear.

Like most developmental transcription factors, FOXC1 is subject to multiple layers of regulation, providing a diversity of opportunities for deregulation in cancer (Figure 2).

### 4.1. Aberrant de-Repression of the FOXC1 Locus

There is compelling evidence demonstrating that *FOXC1* is aberrantly de-repressed in AML and breast cancer. For instance, in normal blood cells the *FOXC1* locus is inaccessible and marked by H3K27me3, indicative of Polycomb repressive complex 2 (PRC2)-dependent repression [40]. Indeed, this lack of *FOXC1* expression is consistently maintained throughout the normal hematopoietic lineage hierarchy, underlining the lack of any cell-intrinsic role in normal hematopoiesis. Furthermore, in K562 leukemia cells which also lack *FOXC1* expression, the *FOXC1* promoter is occupied by the PRC2 subunits EZH2 and SUZ12. These findings contrast with an accessible, active chromatin configuration at *FOXC1* in high-expressing AML cells [68]. Indeed, treatment of normal human CD34^+^ hematopoietic stem and progenitor cells with distinct PRC2 inhibitors was sufficient to achieve a ~9-fold increase in *FOXC1* expression. However, beyond an association between *FOXC1* expression and the t(6,9) translocation or *NPM1* or *FLT3*-ITD mutations, the specific upstream events leading to de-repression of the *FOXC1* locus remain unknown and in need of further investigation [40,68].

Consistent with these findings, unrelated studies also demonstrated that EZH2 maintains repression of *FOXC1* in distinct models of breast cancer [43,87]. In another study, BRCA1 recruits GATA3 to the *FOXC1* promoter to repress its expression in normal breast cells, inhibiting the development of the BLBC subtype of breast cancer. Indeed, forced expression of wild-type BRCA1 in breast cancer cell lines silenced *FOXC1*, whereas GATA3 knockdown induced its expression [88]. This is potentially significant, since *BRCA1* mutations predispose patients to BLBC which often features high *FOXC1* expression [89]. Thus, loss of BRCA1 function by mutation may be a critical driver of *FOXC1* expression in BLBC, although this question has not been directly studied to date. This may also be applicable in ovarian cancer, where BRCA1 mutations are also a known risk factor.

*FOXC1* expression also appears to be deregulated by aberrant promoter demethylation in the context of breast cancer. Lower levels of *FOXC1* promoter methylation correlated with increased mRNA expression and inferior overall survival in one study which encompassed various subtypes of breast cancer [90]. In another study, methylation of the *FOXC1* promoter—and consequently lower levels of mRNA expression—was also found to be higher in tumors positive for expression of the estrogen receptor (ER) [91], consistent with other studies highlighting upregulation of *FOXC1* in ER-negative tumors [42]. While it is likely that methylation of *FOXC1* contributes to aberrant expression patterns elsewhere, this question has not yet been explored in the context of other malignancies.

### 4.2. Growth Factor Receptor Signaling Pathways Influence the Expression of FOXC1

Chronic growth factor receptor signaling is a common hallmark of cancer. Crucially, these pathways are also emerging as recurrent drivers of FOXC1 disruption in several malignancies, either by inducing transcription or by post-translational modification. For instance, in BLBC, high levels of epidermal growth factor receptor (EGFR) expression are a common diagnostic marker, and are significantly correlated with those of FOXC1 in primary BLBC specimens [69]. Furthermore, stimulation of BLBC cells with EGF induces *FOXC1* expression, cell proliferation and migration, whereas knockdown of *FOXC1* abrogated these EGF-dependent effects. Further studies identified the PI3K and MAPK signaling pathways and NF-κB as critical downstream mediators of EGFR-dependent induction of *FOXC1* [92]. NF-κB p65 binds to the *FOXC1* promoter in BLBC cells and activates transcription of a luciferase reporter gene driven by a *FOXC1* promoter fragment. Furthermore, another study found that FOXC1 in turn activates the peptidyl-prolyl isomerase Pin1 which further enhances stability of NF-κB p65 to promote cellular proliferation, invasion and migration [93]. Whether these interesting results from a cell model are also true in other cell settings remains to be determined. 

In addition to EGFR, other studies have implicated distinct growth factor signaling pathways as candidate upstream regulators of *FOXC1*. In one study of FLT3-ITD AML, *FOXC1* was highlighted as an upregulated target gene in primary patient samples [68]. FLT3-ITD receptor mutations cause hyperactivation of MAPK, PI3K and NF-κB signaling pathways, and the establishment of a chromatin signature dominated by RUNX1 and the MAPK-inducible AP-1 family of transcription factors. Most recently, *FOXC1* was identified as a downstream mediator of insulin-like growth factor 1 receptor (IGF1R) signaling in pancreatic cancer, where it promotes EMT and metastasis in vivo [61]. *FOXC1* expression and promoter activity was induced in an IGF-dependent manner in pancreatic cancer cells, and occurred downstream of PI3K pathway activation. Interestingly, FOXC1 seems to also participate in feed-forward activation of *IGF1R*, although the underlying mechanism is not yet clear. A feedback mechanism may also be relevant in hepatocellular carcinoma cells, where induction of *FOXC1* expression by IL-8 seems to further enhance expression of the cognate receptor gene, *CXCR1* [78]. Finally, in unrelated studies of normal vascular endothelial cells, *Foxc1* is activated in a VEGF-signaling dependent manner to increase transcription of arterial specific genes including *Dll4* and *Hey2* [94]. Given that FOXC1 has been observed to upregulate *VEGFA* under tumor hypoxia, it is again possible these molecules constitute a feed-forward regulation loop promoting angiogenesis [77,85].

Interestingly, a role for TGF-β signaling in maintaining repression of *FOXC1* was identified in one study of breast cancer cells. In this context, TGF-β-mediated repression of *FOXC1* reduced transcription of the pro-apoptotic mediator Bim, enhancing breast cancer cell survival [95]. The downstream mediators of FOXC1 repression were found to be SMAD family proteins rather than members of the MAPK pathway, consistent with other studies highlighting the MAPK signaling pathway as an activator of *FOXC1* transcription. Collectively, the available evidence favors generalized roles for MAPK and PI3K signaling in the induction of *FOXC1* expression across cancer types, though the receptors acting upstream of this are likely to be tumor-specific. However, the question of whether these signaling pathways target FOXC1 post-translationally remains incompletely understood. This warrants further investigation, since earlier in vitro studies have shown that MAPK-dependent phosphorylation of FOXC1 at S272 directly increases its cellular half-life and transcriptional activity, and thereby might amplify FOXC1-dependent expression of oncogenes in cancer cells.

### 4.3. Post-Transcriptional and Post-Translational Regulation of FOXC1 in Cancer

In addition to transcriptional mechanisms regulating the abundance of FOXC1 in cancer cells, a number of studies have highlighted post-transcriptional pathways involving microRNAs (miRNAs) or long non-coding RNAs (lncRNAs). For brevity, we have focused on the most well-characterized miRNAs in this review; an extensive catalogue of miRNAs associated with FOXC1 in cancer but with currently unclear in vivo relevance was recently reviewed elsewhere [96]. In studies of endometroid endometrial cancer, a pair of tumor suppressor miRNAs—miR-495 and miR-204—are frequently downregulated in association with elevated FOXC1 expression and tumor progression [49,50]. Both miR-495 and miR-204 interact with the 3′ untranslated region (UTR) of FOXC1 to negatively regulate translation of FOXC1 in vitro, while forced expression of either miRNA led to downregulation of FOXC1 and impaired migration in vitro and/or metastasis in vivo, underlining their functional significance. Another study also reported that miR-204 expression is frequently downregulated in laryngeal squamous cell carcinoma tissues. As in endometrial cancer, this seems to promote progression by amplifying levels of FOXC1 expression.

In addition to being inversely correlated with certain miRNAs, *FOXC1* expression is often correlated with that of a neighboring lncRNA called *FOXCUT* in cancer [62,97,98]. Knockdown of *FOXCUT* is accompanied by a reduction in *FOXC1* expression and FOXC1-dependent oncogenic traits in several malignancies, leading to suggestions that *FOXCUT* stabilizes *FOXC1* transcript to amplify protein translation. This model is consistent with a known function of other lncRNA-mRNA pairs [99], although this has not been functionally tested.

Finally, the potential significance of post-translational regulation of FOXC1 in cancer must not be overlooked. For instance, basic biochemical studies have demonstrated that the stability and *trans*-activating potential of FOXC1 protein are both enhanced in response to MAPK signaling, suggesting that increased activity of these pathways in cancer may potentiate the output of FOXC1 beyond mere upregulation of the *FOXC1* locus [18]. Overall, despite clear indications of potential post-translational deregulation of FOXC1 in cancer, the potential mechanisms at play remain very poorly understood.

## 5. The Utility of FOXC1 as a Cancer Biomarker

Given the prevalence of FOXC1 overexpression in association with an inferior prognosis (Table 1), profiling of FOXC1 expression in primary tumors is increasingly used as an independent prognostic indicator for patients in the clinic. For example, *FOXC1* expression accurately identified patients with the BLBC subtype of breast cancer from other molecular subtypes with comparable sensitivity to more expensive tests such as the PAM50 gene panel in two independent studies of 2341 and 2073 breast cancer cases [100,101]. High *FOXC1* expression further correlates with increased incidence of brain and lung metastases and decreased metastasis-free survival in patients without lymph node involvement [42]. *FOXC1* expression was also associated with activation of a Hedgehog signaling gene signature in primary samples, and resistance to the approved Hedgehog pathway inhibitor vismodegib both in vitro and in vivo. Another study associated FOXC1 expression with downregulation of ERα in breast cancer, potentially accounting for the endocrine resistant nature of primary BLBCs and some relapsed luminal tumors [102].

In AML, high expression of *FOXC1* is associated with adverse prognosis by comparison with FOXC1^lo^ cases [40,67]. More recently, high *FOXC1* expression was significantly correlated with refractoriness to induction chemotherapy and an increased risk of relapse in a combined study of 765 cases [41]. Thus, *FOXC1* expression may represent a predictor of response to treatment that may improve risk-stratification of AML patients. Indeed, there is a strong association between *FOXC1* and tumor staging, metastasis and overall survival in many other cancer types beyond BLBC and AML. Finally, one meta-analysis of several tumor types found that expression of *FOXC1* was more likely to be expressed in late-stage tumors than those at an earlier stage, highlighting the potential association with disease progression [103]. Clearly, classification of tumors by *FOXC1* expression may be a valuable diagnostic tool and potential prognostic indicator for diverse cancer types.

## 6. Conclusions, Current Limitations and Future Prospects

Since *FOXC1* was first described in humans some two decades ago, significant advances have been made in understanding the essential contributions this transcription factor makes to normal development and tissue homeostasis. More recently, the diverse consequences of aberrant *FOXC1* expression in cancer development and progression have begun to emerge. Higher expression of *FOXC1* often correlates with an inferior prognosis across tumor types, with the notable exception of serious ovarian and luminal B breast tumors. A strong body of experimental evidence indicates that FOXC1 generally acts as a proto-oncogene, whose upregulation confers a more tumorigenic disease with greater metastatic potential and therapy resistance. To rationalize the observed disparity between FOXC1’s roles in ovarian/luminal B cancers and other malignancies, future studies should investigate not just the expression levels of FOXC1 but also its sub-cellular localization and activation status. The study highlighting the link between release of FOXC1 protein from nuclear filamins and activation of EMT marker gene expression is likely relevant in this regard [86]. Furthermore, basic studies of FOXC1 structure and function suggest the protein is highly regulated by post-translational modifications [18,19], but this topic remains unexplored in the context of cancer. For example, whilst it is now known that aberrant growth factor receptor signaling generally enhances transcription of *FOXC1* in cancer cells, how these pathways might also influence FOXC1 protein remains unknown. The development of antibodies recognizing FOXC1 protein in specific phosphorylation states would aid clarification of these questions.

Crucially, as FOXC1 is a transcription factor, the diverse, context-specific consequences of aberrant expression are likely to result from the action of distinct target genes activated in each tumor type. However, the current knowledge of bona fide FOXC1 targets—validated with relevant DNA-binding assays—is limited to only a few genes, largely because of a lack of ChIP-grade FOXC1 antibodies. Indeed, studies of AML cells have identified genome-wide changes in chromatin accessibility and TF occupancy in association with *FOXC1* upregulation, suggesting this TF plays a major role in the oncogenic reprogramming of chromatin [67,68]. However, these studies deployed DNase I footprinting assays which, although capable of identifying occupied FOX motifs on a genome-wide basis, are insufficient to formally assign binding events to FOXC1 specifically, especially given the high-level constitutive expression of other Forkhead factors in normal and leukemic hematopoiesis. Overcoming this technical hurdle will enable the genome-wide characterization of FOXC1 action, allowing a more detailed dissection of FOXC1-dependent gene networks and the identification of therapeutically-relevant downstream targets.

From the perspective of developing more efficient therapies for FOXC1-dependent cancers, a more detailed understanding of not just the target genes but also the interacting partners of FOXC1 is needed. Given the observed functional synergy between FOXC1 and other transcription factors in cancer, it is highly likely that such partners exist [40,44], although identification of these partners has so far been limited by the quality of available reagents. Other Forkhead proteins interact with other transcription factors and/or chromatin remodeling enzymes to activate and/or repress gene expression in cancer [104,105].

Crucially, given the high-level expression of other FOX proteins in many cancers, it is not yet clear whether FOXC1 exerts its oncogenic activities mainly by competing with other Forkhead family members for binding, or instead by pioneering distinct sets of binding sites alone or in collaboration with other partners. Historically, transcription factors have been notoriously difficult to target directly from a pharmacological viewpoint, but success has been achieved in some cases by instead targeting the cancer-specific interactions of transcription factors and their co-factors [106,107]. With improved reagents, it may be possible to identify such cancer-specific partners of FOXC1 to aid development of targeted therapies. Finally, for certain cancers it may instead be possible to therapeutically silence expression of *FOXC1* using RNAi-based approaches. One such approach was recently reported in hepatocellular carcinoma cells using a novel nanoparticle-based encapsulation of *FOXC1* siRNA [108], although challenges remain in targeting these nanoparticles to the malignant lesion and ensuring efficient uptake in vivo. Conversely, in the unusual contexts where higher FOXC1 expression promotes an anti-oncogenic phenotype, therapies aimed at raising FOXC1 expression might be an attractive approach to improve outcomes for patients. This concept was recently highlighted in pre-clinical models of breast cancer, where pharmacologic inhibition of EZH2 restored FOXC1 expression and suppressed metastasis specifically in the luminal B subtype [43].

In summary, the oncogenic consequences of aberrant FOXC1 expression are becoming increasingly clear across the breadth of human malignancy. While an understanding of the upstream pathways regulating FOXC1 is beginning to emerge, the downstream mechanisms and target genes driving cancer progression remain largely unclear. Therefore, future efforts to gain a detailed understanding of the molecular functions of FOXC1 in cancer bring promise for the development of novel targeted therapies and improved patient outcomes.

## Figures and Tables

**Table 1:** A summary of FOXC1 alterations in cancer, associated clinical outcomes, and functional consequences of experimental manipulation of *FOXC1* expression.

Cancer Type	*FOXC1* Alteration	Associated Clinical Outcome	Case Sizes	Functional Impact of Experimental *FOXC1* Manipulation	
				Overexpression	Silencing
AML	Overexpression	Inferior OS [40,41]; increased risk of relapse and resistance to induction chemotherapy [41]	270 [40] 765 [41]	Co-operates in vivo with *Hoxa9* to block differentiation and accelerate onset of AML [40]	Loss of clonogenic potential and enhanced morphological differentiation in vitro [40]
Breast (BLBC and Luminal B)	Overexpression	BLBC: Increased metastasis; inferior OS [42]	2073	BLBC: Increased cell growth/survival [42], induced progenitor-like phenotype in vitro [35]; enhanced CSC-like properties in vitro and in vivo [44].	BLBC: Impaired cell proliferation, migration and invasion in vitro [42].
		Luminal B: improved RFS [43]	1142	Luminal B: impaired invasion and lung metastasis formation [43].	-
Colorectal	Overexpression	Increased risk of metastasis; inferior OS/RFS [45,46]	361 [45] 120 [46]	Increased tumorigenicity and metastasis in vivo [45,46]; enhanced Warburg effect in vitro [45].	Impaired tumorigenicity and metastasis in vivo [45,46]; inhibited Warburg effect in vitro [45].
Cervical	Overexpression	Increased risk of invasion, metastasis and relapse; inferior OS [47]	336	-	Suppressed cell growth, migration and invasion in vitro [48].
Endometrial	Overexpression	-	20	Enhanced migration and colony formation in vitro [49,50].	Increased apoptosis in vitro, impaired tumor growth in vivo [49].
Gastric	Overexpression	Inferior OS/RFS [51]	120	-	-
Head and Neck (LSCC and NPC)	Overexpression	LSCC: increased risk of lymph node metastasis [52]	147	-	-
		NPC: increased risk of metastasis [53]	93	-	-
Liver (HCC)	Overexpression	Inferior OS; increased relapse risk [54]	406	Increased cell invasion in vitro and metastasis in vivo [54].	Impaired cell invasion in vitro and metastasis in vivo [54].
Lung (NSCLC)	Overexpression	Inferior OS/RFS [55,56]	125 [55] 1129 [56]	Enhanced CSC-like properties, drug resistance and tumorigenicity in vivo [56].	Suppressed self-renewal and impaired tumorigenicity in vivo [56].
Lymphoma (DLBCL and HL)	Overexpression	DLBCL: trend towards increased extranodal spread [57]	25	-	-
		HL: part of Hodgkin Reed-Sternberg cell-specific gene signature correlated with treatment failure [58]	29	-	-
Melanoma	Overexpression	Inferior OS; increased risk of metastasis [59]	228	Enhanced cell proliferation and invasion in vitro [59]	-
Pancreatic (PDA)	Overexpression	Increased risk of lymph node metastasis, inferior OS [60]	30	Increased cell proliferation and invasion in vitro; promoted tumorigenicity in vivo [61]	Impaired cell proliferation and invasion in vitro [61]
Esophageal (ESCC)	Overexpression	ESCC: increased rate of metastasis, inferior OS [62];	84	ESCC: promoted ESCC cell proliferation, colony formation and invasion in vitro [63]	ESCC: impaired ESCC cell colony formation, and invasion in vitro [62,63]
Osteosarcoma	Overexpression	Higher TNM staging [64]	42	-	Impaired cell proliferation and migration in vitro [64]
Ovarian (Serous)	Overexpression	Improved OS [65]	80	-	-

AML, acute myeloid leukemia; BLBC, basal-like breast cancer; DLBCL, diffuse large B cell lymphoma; ESCC, esophageal squamous cell carcinoma; HCC, hepatocellular carcinoma; HL, Hodgkin lymphoma; LSCC, laryngeal squamous cell carcinoma; NPC, nasopharyngeal carcinoma; NSCLC, non-small cell lung cancer; OS, overall survival; OSCC, oral squamous cell carcinoma; PDA, pancreatic ductal adenocarcinoma; RFS, relapse-free survival; TNM, tumor-node metastasis; –, data either inconclusive or unavailable.
